# Projecting the burden of dental caries and periodontal diseases among the adult population in the United Kingdom using a multi-state population model

**DOI:** 10.3389/fpubh.2023.1190197

**Published:** 2023-09-07

**Authors:** Amal Elamin, John P. Ansah

**Affiliations:** ^1^School of Human Sciences, Faculty of Education, Health and Human Sciences, University of Greenwich, London, United Kingdom; ^2^Center for Community Health Integration, School of Medicine, Case Western Reserve University, Cleveland, OH, United States

**Keywords:** oral diseases, caries, periodontal diseases, system dynamics, United Kingdom, projections

## Abstract

**Objectives:**

With the aging United Kingdom population, oral diseases are expected to increase. Exploring credible projections is fundamental to understanding the likely impact of emerging population-level interventions on oral disease burden. This study aims at providing a credible, evidence-based projection of the adult population in the United Kingdom with dental caries and periodontal diseases.

**Methods:**

We developed a multi-state population model using system dynamics that disaggregates the adult population in the United Kingdom into different oral health states. The caries population was divided into three states: no caries, treated caries, and untreated caries. The periodontal disease population was disaggregated into no periodontal disease, pocketing between 4 and < 6 mm, 6 and < 9 mm, and 9 mm or more. Data from the 2009 dental health survey in the United Kingdom was used to estimate age and gender-specific prevalence rates as input to the multi-state population model.

**Results:**

Of the population 16 years and older, the number with carious teeth is projected to decrease from 15.742 million in the year 2020 to 15.504 million by the year 2050, representing a decrease of 1.5%. For individuals with carious teeth, the older adult population is estimated to constitute 62.06% by 2050 and is projected to increase 89.4% from 5.079 million in 2020 to 9.623 million by 2050. The adult population with periodontal pocketing is estimated to increase from 25.751 million in 2020 to 27.980 million by 2050, while those with periodontal loss of attachment are projected to increase from 18.667 million in 2020 to 20.898 million by 2050. The burden of carious teeth and periodontal diseases is anticipated to shift from the adult population (16–59 years) to the older adult population. The older adult population with carious teeth is estimated to rise from 32.26% in 2020 to 62.06% by 2050, while that for periodontal disease is expected to increase from 42.44% in 2020 to 54.57% by 2050.

**Conclusion:**

This model provides evidence-based plausible future demand for oral health conditions, allowing policymakers to plan for oral health capacity to address growing needs. Because of the significant delay involved in educating and training oral health personnel, such projections offer policymakers the opportunity to be proactive in planning for future capacity needs instead of being reactive.

## Introduction

Oral diseases are among the most prevalent non-communicable diseases (NCD) globally, affecting 3.5 billion people in 2019 (1). They encompass a range of diseases and conditions that include dental caries, periodontal disease, tooth loss, oral cavity cancer, dental trauma, noma, and congenital anomalies such as cleft palate and lip (2). The estimated number of cases of oral diseases globally is approximately 1 billion more than the combined number of cases of the five main NCDs: cardiovascular disease, diabetes mellitus, chronic respiratory diseases, mental disorders, and cancers (2). Among these oral diseases, dental caries in permanent teeth and severe periodontitis are the most common and major causes of tooth loss. Untreated dental caries in permanent teeth stand out as the most prevalent disease on a global scale, with 2.3 billion people having it, followed by severe periodontitis, which affects approximately 1 billion people globally (3). Furthermore, oral diseases impose a high economic burden and are the fourth most expensive group of diseases to treat globally (4). In 2015, the estimated direct and indirect costs of oral diseases amounted to $356.80 and $187.61 billion, respectively, totaling the global economic burden of oral diseases to $544.41 billion (5). Despite oral diseases being wildly prevalent, largely preventable, and having a substantial economic burden, they are rarely prioritized in global health policy.

In the United Kingdom, the prevalence trends of untreated caries indicate a sharp decline in dental caries among adults between 1998 and 2009, from 54 to 31% (6). In contrast, trends in periodontal status vary, with only 17% of British adults having healthy periodontal status. More severe periodontal disease among British adults increased from 6% in 1998 to 9% in 2009, while mild and moderate periodontal disease affecting 37% of adults has decreased (6). Despite these trends, the expected increase in the aging population and other demographic shifts are anticipated to increase the cumulative burden of oral diseases substantially. The projected increase in the burden of oral diseases is supported by epidemiological evidence. Between 1990 and 2019, there was a significant global increase in estimated cases of oral diseases, surpassing 1 billion, representing a 50% increase (2, 7). This increase was higher than the population growth of around 45% during the same period. Furthermore, in high-income countries, the case numbers for oral diseases rose by 23%, outpacing demographic growth in those countries (7).

In recent years, there has been growing interest in oral health burden projections, which have shown to be valuable in estimating future trends and informing public health policies. Nevertheless, the application of projection analysis in oral health research remains limited and scattered in scope, projection method, and target population, particularly when considering the utilities this approach offers. Few studies have projected the prevalence of dental caries and caries-free in primary and permanent dentitions among different age groups, as well as projections of other oral conditions such as edentulism and oral cancers. Jordan et al. (8) projected trends in dental caries in permanent dentition among children aged 12 years, adults aged 35–44 years, and older adults aged 65–74 years in Germany until 2030 using log-linearization and a linear regression model. The authors reported decreases in the cumulative caries experience from 1.1 billion DMFT in 2000 to 867 million in 2015 and projected a further decrease to 740 million in 2030 (8). Conversely, a projection analysis was conducted in Thailand and used a system dynamics model to forecast dental caries in permanent teeth among adults and older adults (≥ 15 years old) until 2040 under different policy options (9). The study projected an increase in dental caries experiences among Thai adults and older due to the aging population (9). Furthermore, caries experience among younger age groups was examined in a 2017 study where a shorter-term-projection analysis using an autoregressive integrated moving average (ARIMA) model and a gray predictive model (GM) to forecast early childhood caries prevalence among children aged 5 years from 2014 to 2018 was conducted in China (10). While in Malaysia, the caries-free prevalence among schoolchildren aged 6, 12, and 16 years old was projected from 2020 to 2030 using three time-series models: double exponential smoothing (DES), autoregressive integrated moving average (ARIMA), and the error, trend, and seasonal (ETS) model, and reported that, caries-free prevalence to increase steadily in 6- and 12-year-old schoolchildren from 2020 to 2030 (11). Projection analyses have also been conducted in other oral health conditions such as edentulism and oral and oropharyngeal cancer mortalities (12, 13). Schwendicke and colleagues used Monte Carlo simulations to forecast the prevalence of tooth loss among older adults (aged 65–74 years) in Germany until 2030 (13). Infante Cossio et and colleagues used the Nordpred program to generate a predictive model to predict Oral cavity cancer (OCC) and oropharyngeal cancer (OPC) mortality rate in Spain until 2044 (12). The predictive model projected a higher mortality rate in females than in males for OCC in the period 2040–2044, while deaths for OPC were projected to decrease in males and gradually increase in females (12). However, none of these studies were conducted in the United Kingdom, attempted to project the burden of periodontal diseases or incorporated the demographic changes into projecting the burden of caries and periodontal disease, clearly indicating a gap.

In the context of rapid population aging, in the United Kingdom, it is projected that the number of people aged 85 years and above will increase from 1.7 million in 2020 (2.5% of the United Kingdom population) to an estimated 3.1 million by 2045 (4.3% of the United Kingdom population) (14). To manage the future demands of this aging population, it is crucial to quantify the burden of major oral diseases. This will help estimate and optimize resource allocation for prevention and treatment needs. Additionally, it will aid in planning future demands for dental health services, capacity planning, and workforce requirements. This study aims to use a multi-state population model to project the burden of dental caries and periodontal diseases among the adult population in the United Kingdom and to provide evidence to support population-level intervention evaluation.

## Methods

Based on the 2009 dental health survey (ADHS) data from the United Kingdom, the systems science methodology of System Dynamics was used to develop a multi-state population simulation model (15–18) for projecting the adult population of the United Kingdom with dental caries and periodontal diseases. System dynamics models consist of interacting sets of differential, and algebraic equations developed from a broad range of relevant empirical data (19, 20). The simulation models developed are used to understand the underlying dynamics, complex systems, or structures that cause the problems. The system dynamics method has been used to address complex health issues in healthcare (21–24). There is limited application of the system dynamics method in oral health (25–28), and this research study adds to the limited application to demonstrate its utility to oral public health.

### Model structure

The oral health model consists of two sub-models: dental caries sub-model and the periodontal diseases sub-model. The periodontal disease sub-model was further divided into two sub-models (a) the periodontal pocketing sub-model and (b) the periodontal loss of attachment (LOA) sub-model. The oral health model presented herein was developed as follows: first, a validated dynamic multi-state population model that simulates outcomes of interest using available data and information from literature was developed. Next, the multi-state population model was presented to clinician scientists with expertise in dentistry to verify the model structure and its assumptions regarding causal relationships and its consistency with existing evidence. The model was refined in an iterative process until it was considered adequate concerning its realism, clarity, and ability to capture important issues of interest to the purpose of the model. Following the experts’ review and revision of the model structure consistency with experts’ knowledge, the model was parameterized, and simulated to generate evidence-based projections of dental caries and periodontal diseases in the United Kingdom.

### Dental caries sub-model

**Figure 1:**
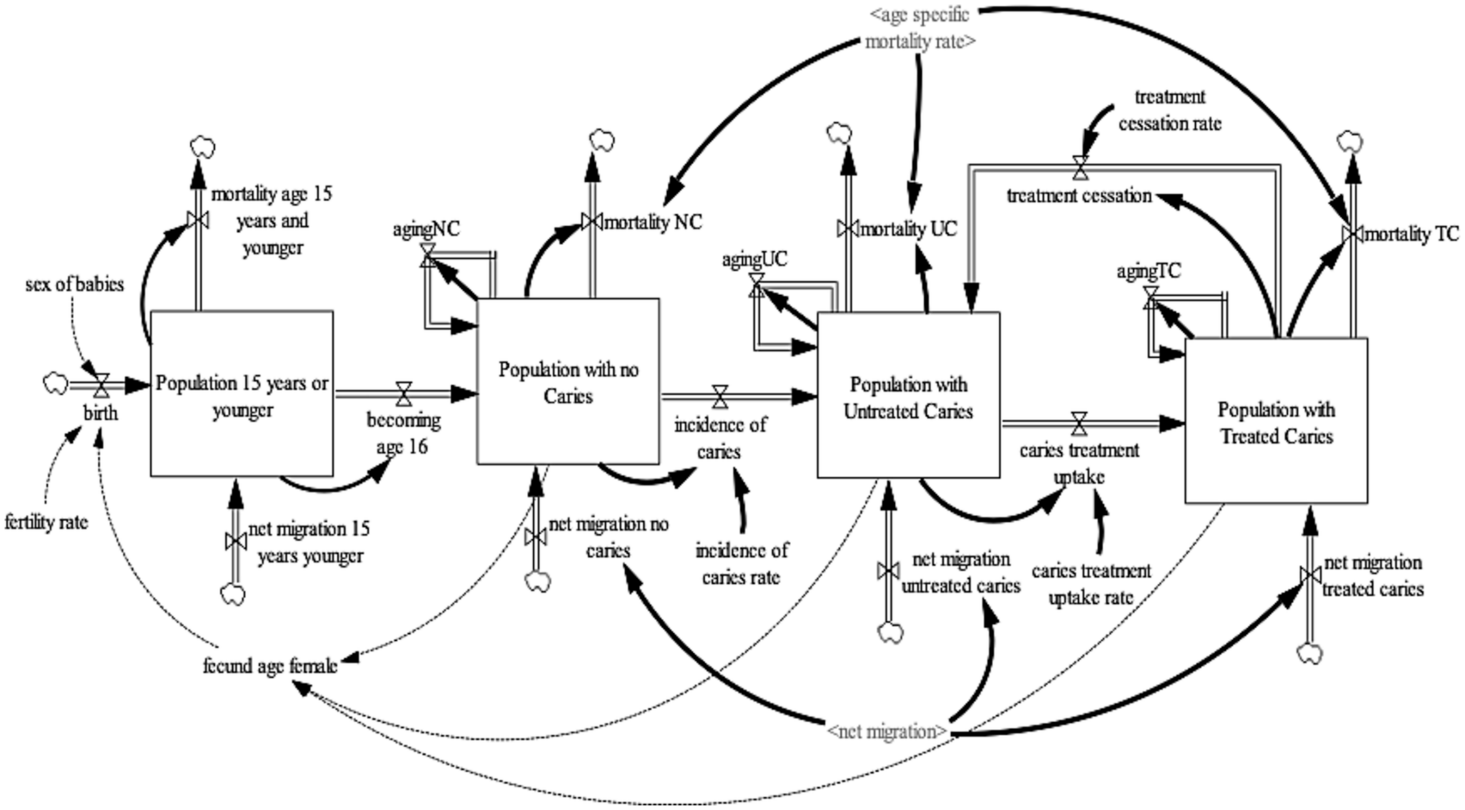
Caries sub-model.

The caries sub-model (Figure 1) projects the United Kingdom adult population (age 16 years and older) with carious teeth. To project the United Kingdom adult population with carious teeth, the United Kingdom population 16 years and older was disaggregated into three health states—no-caries, untreated caries, and treated caries. These health states were further disaggregated by age (single age cohorts from age 16 to age 100 and older) and gender (male, female). For the purpose of this model, the “no caries” health state refers to individuals with teeth with no visible decay or restoration of any kind, including those such as veneers and crowns, which are not always placed to manage the disease (29). It also includes teeth with sealants that were sound or fractured but with no evidence of caries (29). The “untreated caries” health state refer to individuals with teeth with visual caries or cavitated caries or teeth that were so broken down, possibly with pulpal involvement, that they were unrestorable (29). It includes teeth that had restorations with recurrent caries (29). Lastly, “treated caries” health state refers to individuals with teeth in which a filling has been placed but which are now sound with no active decay and no damage to the filling (29). To ensure consistency and validity of the model output, an additional state that accounts for the population age 15 years or younger was included. This ensures that individuals aged 15 transitions to the adult population with no caries health state. To establish a consistent aging process, the population aged 15 years or younger was divided into single age cohorts (age 0–15 years).

The population 15 years or younger increases through births and net migration and decreases by mortality and becoming age 16. Births were estimated by fecund female population (age 15–49) and fertility rate (30); while net migration is estimated by calibration. Likewise, mortality is determined by age-gender-specific mortality rates from life tables (31). At the end of each year, the surviving population in each age cohort flows to the subsequent cohort, except the final age cohort, age 100 and older. The population with no-caries increases with individuals becoming age 16 and net migration of individuals with no-caries and decreases via incidence of caries and mortality from the population with no-caries. The incidence rate of caries development is estimated by calibration. The population with untreated cries increases by the incidence of caries, net migration of individuals with untreated caries, and caries treatment cessation of individuals with treated caries; and decreases by caries treatment uptake and mortality among the population with untreated caries. Caries treatment uptake rate and caries treatment cessation rate are estimated *via* calibration. Lastly, the population with treated caries increases by caries treatment uptake and net migration of individuals with treated caries and decreases by caries treatment cessation and mortality among the population with treated caries.

### Periodontal disease sub-model

**Figure 2:**
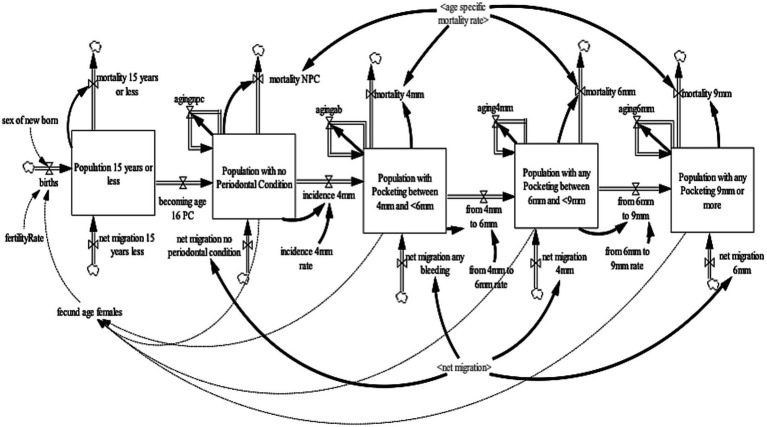
Periodontal pocketing sub-model.

**Figure 3:**
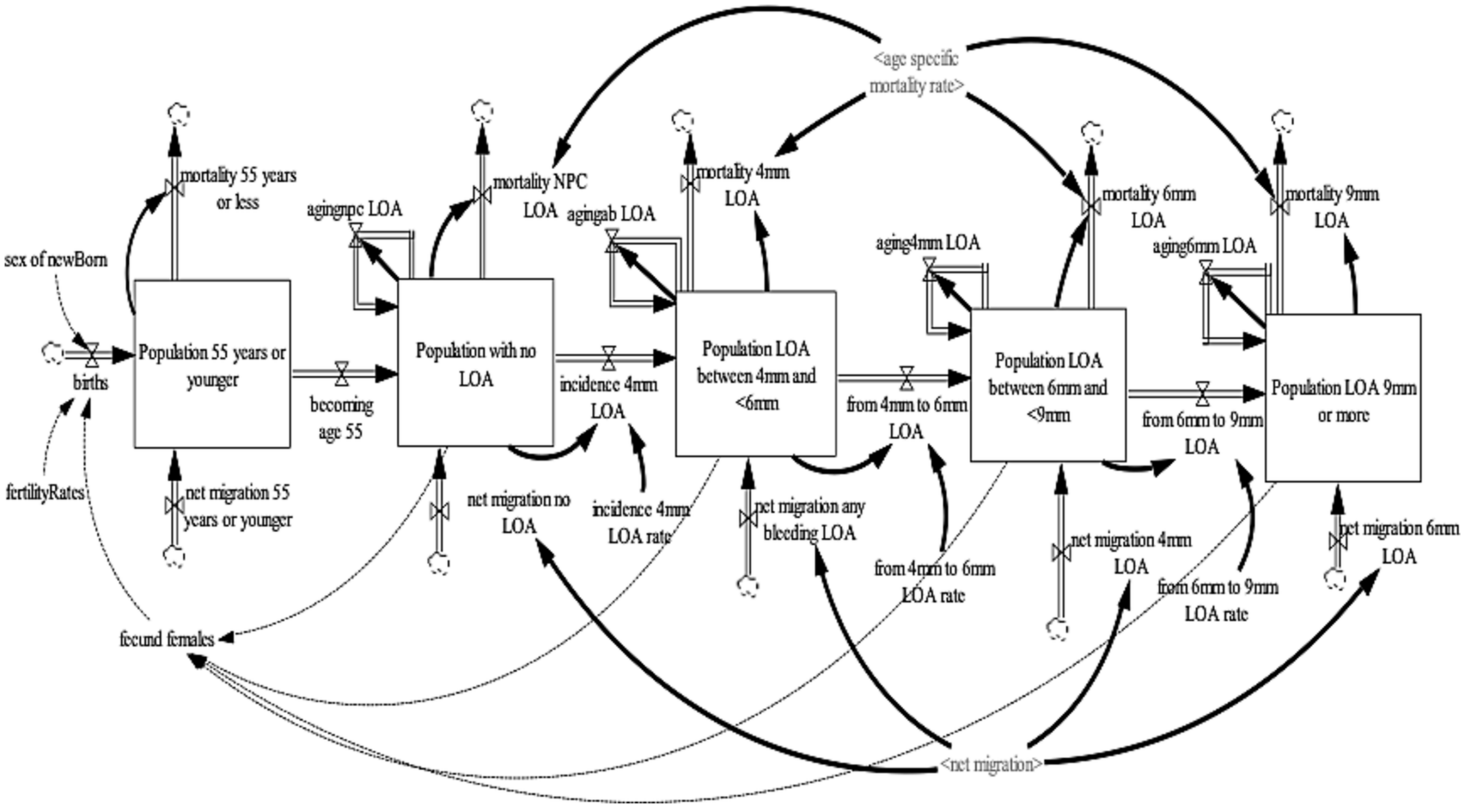
Periodontal loss of attachment sub-model.

The periodontal diseases sub-model projects the United Kingdom adult population (age 16 years and older) with periodontal diseases. The periodontal disease sub-model was further divided into two sub-models (a) the periodontal pocketing sub-model (Figure 2) and (b) the LOA sub-model (Figure 3). For the periodontal pocketing sub-model, the adult population of the United Kingdom was disaggregated into four health states—no-periodontal condition, any pocketing 4 to <6 mm, any pocketing 6 to <9 mm, and any pocketing 9 mm or more—to project the periodontal disease in the United Kingdom. These health states were further disaggregated by age (single age cohorts from age 16 to 100 and older) and gender (male, female). For the purpose of this model, periodontal pocketing is defined as a pathologically deepened gingival sulcus measured from the gingival margin to the base of the pocket (29). Pockets deeper than 3.5 mm were recorded to give an indication of disease and are reported here at thresholds of 4, 6, and 9 mm. The 4, 6, and 9 mm pockets can be classified as mild, moderate, and severe periodontal pocketing, respectively (29). To ensure consistency and validity of the model output, an additional state that accounts for the population aged 15 years or younger were included to ensure that individuals aged 15 transitions to the adult population with a no-periodontal condition health state. To ensure a consistent aging process, the population aged 15 years or younger was divided into single age cohorts (age 0–15 years).

The population of 15 years or younger increases through births and net migration and decreases by mortality and age 16. Births were estimated by fecund female population (age 15–49) and fertility rate (30), while net migration was estimated by calibration. Likewise, mortality is determined by age-gender-specific mortality rates from life tables (31). At the end of each year, the surviving population in each age cohort flows to the subsequent cohort, except the final age cohort, age 100 and older. The population with the no-periodontal condition increases as individuals become age 16 years and the net migration of individuals with the no-periodontal condition and decreases by the incidence of 4 mm pocketing and mortality of the population with the no-periodontal condition. The population with pocketing 4 to <6 mm increases by the incidence of 4 mm pocketing and net migration of individuals with 4 to <6 mm pocketing and decreases by the transition from 4 to 6 mm pocketing and mortality of the population with pocketing 4 to <6 mm. The population with pocketing 6 to <9 mm increases by the transition from 4 to 6 mm pocketing and net migration of individuals with 6 to <9 mm pocketing and decreases by the transition from 6 to 9 mm pocketing and mortality of the population with pocketing 6 to <9 mm. Lastly, the population with any pocketing of 9 mm or more increases by the transition from 6 mm to 9 mm pocketing and net migration of individuals with 9 mm pocketing and decreases by the mortality of the population with pocketing of 9 mm or more.

In the 2009, ADHS LOA was only assessed for subjects aged 55 years old or over (29). Therefore, the LOA sub-model projects individuals 55 years or older with periodontal LOA in the United Kingdom. The LOA sub-model was disaggregated into four health states—no-loss of attachment, LOA 4 to <6 mm, LOA 6 to <9 mm, and LOA 9 mm or more. These health states were further disaggregated by age (single age cohorts from age 16–100 and older) and gender (male, female). To this model, loss of attachment is defined as damage over a lifetime that takes into account gum recession (which will often occur alongside pocketing). It is measured with the periodontal probe as the distance from the cementoenamel junction (CEJ) to the base of the pocket. As with periodontal pocketing, the worst score for each sextant was recorded and the thresholds of 4, 6, and 9 mm were used, and were classified as mild, moderate, and severe LOA, respectively (29, 31, 32). To ensure consistency and validity of the model output, an additional state that accounts for the population age 54 years or younger were included to ensure that individuals aged 54 transitions to the population 55 years and older with a no-LOA health state. To ensure a consistent aging process, the population aged 54 years or younger was divided into single age cohorts (age 0–54 years).

The population 54 years or younger increases through births and net migration and decreases by mortality and becoming age 55. Births were estimated by fecund female population (age 15–49) and fertility rate (30); while net migration is estimated by calibration. Likewise, mortality is determined by age-gender-specific mortality rates from life tables (31). At the end of each year, the surviving population in each age cohort flows to the subsequent cohort, except the final age cohort, age 100 and older. The population with the no-LOA increases as individuals become age 55 years and the net migration of individuals with no-LOA and decreases by the incidence of 4 mm LOA and mortality of the population with the no-loss of attachment. The population with a LOA of 4 to <6 mm increases by the incidence of 4 mm LOA and net migration of individuals with 4 to <6 mm LOA and decreases by the transition from 4 to 6 mm LOA and mortality of the population with 4 to <6 mm loss of attachment. The population with a LOA of 6 to <9 mm increases by the transition from 4 to 6 mm LOA and net migration of individuals with 6 to <9 mm LOA and decreases by the transition from 6 to 9 mm LOA and mortality of the population with LOA 6 to <9 mm. Lastly, the population with a LOA of 9 mm or more increases by the transition from 6 to 9 mm LOA and net migration of individuals with 9 mm or more LOA and decreases by the mortality of the population with LOA of 9 mm or more.

### Model assumptions

Birth rate and age-gender-specific mortality rates were assumed to be constant over the simulation time. However, it is important to emphasize that these variables were included in the sensitivity analysis and were varied to evaluate their impact on outcomes of interest. Individuals becoming age 16 were assumed to transition directly into the population with no-caries, while a similar assumption was made for individuals 16 years transitioning to no-periodontal condition. For the caries sub-model, the incidence rate of caries, caries treatment uptake rate, and caries treatment cessation rate were assumed to be constant across age. Similarly, for the periodontal sub-model, the incidence rate of 4 mm pocketing, the transition from 4 to 6 mm pocketing, and the transition from 6 to 9 mm pocketing rates were assumed to be constant across age. Also, for the LOA sub-model, the incidence rate of 4 mm loss of attachment, the transition from 4 to 6 mm loss of attachment, and the transition from 6 to 9 mm LOA rates were assumed to be constant across age. However, all these parameters were included in the sensitivity analysis.

### Data

The oral health model used demographic data in the United Kingdom and the 2009 Adult Dental Health Survey (ADHS) data as input for the model, and was accessed with permission from the United Kingdom Data Service (33). The demographic datasets used as input for the sub-models were obtained from the Office of National Statistics (30, 31, 34). Data regarding the prevalence of caries and periodontal conditions were obtained from the 2009 ADHS data (29). The ADHS is a cross sectional study conducted every 10 years. The survey provides oral health status of the population and their access to, and experience of, dental services. The 2009 ADHS comprised of two components: a questionnaire survey and a clinical survey, and used two-stage cluster sampling design, with a sample size of 13,400 households. The 13,400 households comprised 1,150 households from each of the 10 English Strategic Health Authorities, 1,150 from Wales, and 750 from Northern Ireland. The participating households, all adults 16 years or older, were invited for face-to-face interview and individuals with at least one natural tooth were invited to undergo a subsequent dental examination, conducted by NHS salaried dentist who attended study training over 4 days (29, 35). The list of model input parameters is provided in the Appendix A.

### Model validation and sensitivity analysis

The typical structure and behavior test (36, 37) of system dynamics models were applied to validate the oral health model. For the validity of the model structure, the oral health model was presented to two clinician-scientists with expertise in dentistry to verify the model structure and its assumptions regarding causal relationships and its consistency with existing literature evidence. Consequently, we are confident that the model is grounded on current knowledge and evidence on the development and progression of caries and periodontal diseases. The behavior test compared simulated model outcomes with available data. Since the 2009 ADHS data is cross-sectional data, we generated a time series data of the prevalence of caries and periodontal diseases and used it to calibrate the incidence and transition rates in caries and periodontal disease sub-models. The time series data was generated by multiplying the age-specific prevalence rates by a validated dynamic population model of the United Kingdom. Appendix B shows the simulation of caries and periodontal diseases as compared to available data. The results suggest that the simulation model output compares favorably with data, indicating that the model performs credibly for the visual fit test.

For the sensitivity analysis, a two-way sensitivity analysis was performed to evaluate the impact of a change in selected model parameters on the outcomes of interest. The parameters included in the sensitivity analysis, where they were varied simultaneously are fertility rate, net migration rate, age-specific mortality rate, incidence of caries rate, caries treatment uptake rate, caries treatment cessation rate, incidence 4 mm pocketing rate, transition from 4 to <6 mm pocketing rate, transition from 6 to <9 mm pocketing rate, incidence of 4 mm LOA rate, transition from 4 to <6 mm LOA rate, and transition from 6 to <9 mm LOA rate. These parameters were varied simultaneously by ±50%, and the model was simulated 500 times. The estimated average and the minimum and maximum values at a 95% confidence interval were used to show the uncertainty around the projected outcomes.

## Results

Table 1 shows the results of the projected number of adult 16 years and older with dental caries in the United Kingdom from 2020 to 2050. The number of people in the United Kingdom 16 years and older is projected to increase from 54.709 million (with a 95% confidence interval of 54.670–54.747) in 2020 to 58.356 million (57.804–58.907) by 2050, representing a 6.7% (5.7–7.6%) increase. Due to population aging, the older adult population 60 years and older is projected to increase the most by 33.4% (33.3–33.5%) from 16.884 million (16.803–16.965) in 2020 to 22.526 million (22.404–22.648) by 2050. Due to the low fertility rate, the population of 16–24 years and 25–59 years are projected to decline by 7.7% (10.7–4.7%), and 4.7% (6.1–3.4%) respectively, from the year 2020 to 2050.

**Table 1:** Projected number of adult 16 years and older with dental caries in the United Kingdom from 2020 to 2050.

Age cohort	2020	2035	2050	Relative change (2020–2050) %
Population (million)				
16–24 years	6.879 [6.868–6.889]	6.984 [6.817–7.151]	6.349 [6.134–6.564]	−7.7% [−10.7-(−4.7)]
25–59 years	30.945 [30.913–30.977]	30.177 [30.049–30.305]	29.480 [29.025–29.934]	−4.7% [−6.1-(−3.4)]
60+ years	16.884 [16.803–16.965]	21.093 [20.972–21.215]	22.526 [22.404–22.648]	33.4% [33.3–33.5]
Total	54.709 [54.670–54.747]	58.255 [58.079–58.432]	58.356 [57.804–58.907]	6.7% [5.7–7.6]
Caries (million)				
16–24 years	0.335 [0.329–0.340]	0.244 [0.234–0.255]	0.232 [0.219–0.244]	−30.7% [−33.3-(−28.2)]
25–59 years	10.327 [10.281–10.373]	8.241 [8.143–8.338]	5.649 [5.490–5.808]	−45.3% [−46.6-(−44.0)]
60+ years	5.079 [5.055–5.104]	7.863 [7.795–7.931]	9.623 [9.514–9.731]	89.4% [88.2–90.7]
Total	15.742 [15.666–15.818]	16.349 [16.174–16.525]	15.504 [15.224–15.784]	−1.5% [−2. 8-(−0.2)]
Untreated caries (million)				
16–24 years	0.191 [0.186–0.195]	0.193 [0.184–0.201]	0.182 [0.173–0.192]	−4.4% [−7.3-(−1.5)]
25–59 years	2.687 [2.654–2.720]	3.359 [3.292–3.426]	3.559 [3.448–3.670]	32.4% [29.9–34.9]
60+ years	1.399 [1.381–1.417]	2.758 [2.712–2.804]	3.755 [3.686–3.823]	168.3% [166.9–169.7]
Total	4.278 [4.222–4.334]	6.311 [6.189–6.432]	7.497 [7.308–7.686]	75.2% [73.1–77.3]
Treated caries (million)				
16–24 years	0.143 [0.142–0.145]	0.0517 [0.0493–0.0540]	0.0492 [0.0465–0.0520]	−65.7% [−67.3-(−64.1)]
25–59 years	7.639 [7.627–7.652]	4.881 [4.851–4.911]	2.089 [2.041–2.137]	−72.6% [−73.2-(−72.1)]
60+ years	3.680 [3.673–3.686]	5.104 [5.083–5.126]	5.868 [5.827–5.908]	59.4% [58.6–60.3]
Total	11.464 [11.443–11.484]	10.038 [9.984–10.092]	8.006 [7.915–8.098]	−30.2% [−30. 8-(−29.5)]

Of the population 16 years and older, the number with carious teeth is projected to decrease from 15.742 million (15.666–15.818) in the year 2020 to 15.504 million (15.224–15.784) by the year 2050, representing a decrease of 1.5% (2.8–0.2%). For individuals with carious teeth, the older adult population is estimated to constitute 62.06% by 2050 and is projected to increase 89.4% (88.2–90.7%) from 5.079 million (5.055–5.104) in 2020 to 9.623 million (9.514–9.731) by 2050. The individuals between ages 16 and 24 years with carious teeth are projected to decrease from 0.335 million (0.329–0.340) in 2020 to 0.232 million (0.219–0.44) by 2050, which represents a decrease of 30.7% (33.3–28.2%). Likewise, the number of individuals between the ages 25–59 years with carious teeth is projected to decrease from 10.327 million (10.281–10.373) in 2020 to 5.649 million (5.490–5.808) by 2050, representing a decrease of 45.3% (46.6–44.0%). Of the individuals with carious teeth, the majority are estimated to be treated. The untreated carious teeth are expected to increase from 4.278 million (4.222–4.334) in 2020 to 7.497 million (7.308–7.686) by 2050, representing a 75.2% (73.1–77.3%) increase.

Table 2 shows the results of the projected number of adult 16 years and older with periodontal diseases in the United Kingdom from 2020 to 2050. The number of individuals with mild pocketing (pocketing between 4 and < 6 mm) is projected to increase from 20.676 million (20.552–20.799) in 2020 to 22.495 million (22.036–22.954) by 2050, representing 8.8% (7.2–10.4%) increase, while those with moderate pocketing (pocketing between 6 and < 9 mm) is estimated to decrease from 3.162 million (3.151–3.173) in 2020 to 2.489 million (2.440–2.537) by 2050, which is a decrease of 21.3% (22.6–20.0%). However, the number of people with severe pocketing (pocketing which is 9 mm or more) is projected to increase 56.7% (54.1–59.2%) from 1.912 million (1.883–1.941) in 2020 to 2.996 million (2.902–3.090) by 2050. For individuals with pocketing, by 2050, the older adult population 60 years and older is projected to constitute 54.6% [which is 15.269 million (15.062–15.475)] and is expected to increase by 39.7% (38.4–41.1%) from the year 2020 to 2050.

**Table 2:** Projected number of adult 16 years and older with periodontal diseases in the United Kingdom from 2020 to 2050.

Age cohort	2020 (Projection in million)	2035 (Projection in million)	2050 (Projection in million)	Relative change (2020–2050) %
Periodontal pocketing^*^				
16–24 years	0.674 [0.659–0.690]	0.664 [0.636–0.693]	0.629 [0.595–0.662]	−6.7% [−9.7-(−4.0)]
25–59 years	14.147 [14.942–14.252]	12.955 [12.733–13.177]	12.082 [11.720–12.444]	−14.6% [−16.5-(−12.7)]
60+ years	10.929 [10.886–10.971]	14.068 [13.946–14.190]	15.269 [15.062–15.475]	39.7% [38.4–41.1]
Total	25.751 [25.588–25.913]	27.689 [27.316–28.061]	27.980 [27.379–28.582]	8.7% [7.0–10.3]
Mild pocketing	20.676 [20.552–20.799]	22.234 [21.953–22.516]	22.495 [22.036–22.954]	8.8% [7.2–10.4]
Moderate pocketing	3.162 [3.151–3.173]	2.867 [2.839–2.894]	2.489 [2.440–2.537]	−21.3% [−22.6-(−20.0)]
Severe pocketing	1.912 [1.883–1.941]	2.587 [2.523–2.650]	2.996 [2.902–3.090]	56.7% [54.1–59.2]
LOA^**^				
Mild LOA	13.505 [13.448–13.562]	15.037 [14.975–15.100]	15.666 [15.591–15.740]	16.0% [15.9–16.1]
Moderate LOA	4.352 [4.294–4.411]	4.398 [4.290–4.507]	4.278 [4.149–4.406]	−1.7% [−3.4-(−0.1)]
Severe LOA	0.809 [0.798–0.820]	0.916 [0.895–0.938]	0.954 [0.928–0.980]	18.0% [16.3–19.6]
Total	18.667 [18.542–18.792]	20.353 [20.164–20.541]	20.898 [20.672–21.124]	12.0% [11.5–12.4]

* Periodontal pockets severity is classified as follows: mild pocketing between 4 and < 6 mm; moderate pocketing between 6 and < 9 mm; and severe pocketing ≥ 9 mm.

** Loss of attachment (LOA) severity is classified as follows: mild LOA between 4 mm and < 6 mm; moderate LOA between 6 and < 9 mm; and severe LOA ≥ 9 mm.

The number of individuals with periodontal LOA is projected to increase from 18.6767 million (18.542–18.792) in the year 2020 to 20.898 million (20.7672–21.124) by 2050. Of the individuals with LOA, mild LOA is projected to increase from 13.505 million (13.448–13.562) in the year 2020 to 15.666 million (15.591–15.740) by 2050. The number of people with moderate LOA is projected to decrease from 4.352 million (4.294–4.411) in the year 2020 to 4.278 million (4.149–4.406) by 2050. Lastly, the number of people with severe LOA is projected to increase from 0.809 million (0.798–0.820) by the year 2020 to 0.954 million (0.928–0.980) by 2050.

## Discussion

A multi-state population model using system dynamics was used to provide, for the first time, a projection of the adult population in the United Kingdom with dental caries and periodontal diseases from 2020 to 2050. The results from the oral diseases burden simulation model show that while the burden of carious teeth is projected to decrease from 2020 to 2050, the periodontal disease burden is projected to increase. However, due to population aging, the older adult population (≥60 years old) is expected to experience the highest burden of carious teeth and periodontal diseases, while a significant decrease in burden is projected from individuals between the ages of 16 and 59 years.

The insights from this 30 years projection are as follows: First, the burden of carious teeth and periodontal diseases is anticipated to shift from the adult population (16–59 years) to the older adult population. The older adult population with carious teeth is estimated to rise from 32.26% in 2020 to 62.06% by 2050. Our forecasting analysis indicating a demographic shift of caries morbidity is in line with findings from a study conducted in Germany, which assessed the trends in dental caries experience in the permanent dentition from 1997 to 2014 and projected caries experience to 2030 (8). The authors reported in their 2030 projection that young seniors (aged 65–74 years) and seniors over 75 years are expected to experience an increase in decayed and filled teeth to some degree, mainly due to the retention of more teeth that are now at risk for caries, while decayed teeth and filled teeth are projected to decrease in younger age groups (8). In terms of decreasing trends of untreated caries among the 16–24 years age group, our findings are in agreement with, those reported by Urwannachotima and colleagues, who reported that the proportion with untreated dental caries is expected to decrease slightly over the simulation time (9). They suggested that the observed increase in dental caries could be explained by the increasing and aging population, which is comparable to our findings (9). Second, the older adult population with periodontal disease is expected to increase from 42.44% in 2020 to 54.57% by 2050. The majority of periodontal diseases (80.39%) are projected to remain as mild periodontal diseases. Lastly, untreated carious teeth are estimated to increase by 75.2% from 2020 to 2050. However, the lack of United Kingdom based projections for dental caries and periodontal diseases, hinders any meaningful comparisons. These are important issues to be addressed in future research. These insights from this study has implications for public health, oral health, and economics. In terms of public health, these insights highlight the importance of continued education and improving health of the public on the causes of oral diseases and actions individuals can take to reduce the risk of developing them. Additionally, they emphasize the need for active engagement with stakeholders to explore innovative ways to address social determinants of health that negatively impact oral health outcomes, as well as the implementation of preventive oral health systems will be needed (38), as socio-behavioral and environmental factors play a significant role in oral disease and health (39–41). As for oral health, this study provides evidence-based projections of plausible future demand for oral health conditions, enabling policymakers to plan for oral health capacity to address the growing needs. By adopting this approach, policymakers can proactively plan for future capacity needs instead of being reactive, which often entails substantial delays in educating and training oral health personnel. Despite the scarcity of studies forecasting oral diseases among adults, a research study has used evidence-based projections of plausible future demand for oral health conditions. The study projected the prevalence of edentulism and its occurrence in 2030 among older Germans (aged 65–74 years) using Monte Carlo simulations. It emphasized the importance of accounting for demographic dynamics in the projection and using credible projections for oral diseases (13). Furthermore, the insight that most of the oral disease burden is projected to affect the older adult individuals, oral healthcare services should pay particular attention to the oral health needs of this population group. Proactively planning to address these projected needs can prevent the reduced quality of life associated with oral diseases (42). In the United Kingdom, there are substantial concerns about oral health inequalities evident across the social spectrum and life course, mainly reflecting socio-economic inequalities in overall health (43, 44). With the anticipated increase in dental caries and periodontal diseases among the older adult population and the rapid population aging, this age-related inequality is expected to become more challenging. Therefore, it should have a greater urgency to address risks that could exacerbate these inequalities (43, 44) If not addressed, it could lead to a decline in the oral health of the population, impeding progress toward achieving long-term health goals for the country. The economic burden associated with oral diseases burden includes the direct cost of treatment, the indirect cost of productivity losses due to absence from work and school, and intangible costs such as pain, the problem with biting, chewing, and eating, and the expression of emotions such as smiling (45). Consequently, policymakers and health systems have the opportunity to prioritize and implement cost-effective interventions that have the potential to reduce the economic cost related to the oral health burden. In the United Kingdom, oral health inequalities are a significant concern that has been extensively documented (46, 47).

The study findings hold significant importance, given the ongoing challenges facing oral healthcare system in United Kingdom (48–51). The oral health system has been struggling to meet the growing oral health needs of the population, especially post-pandemic (48). Dental services have become increasingly limited and strained due to workforce shortages, NHS budget cuts, and an increasing number of older patients (48–51). Unless these challenges are promptly addressed and effectively responded to, the projected oral health picture will worsen. These challenges highlight the need for increased attention and investment in oral health care and integration into the broader healthcare framework to accommodate aging populations and their economic burdens of dental diseases.

One of the main limitations of this study is the assumption that the 2009 ADHS age-specific prevalence rates of caries and periodontal disease remains unchanged over the simulation time. Future studies should use current representative studies to improve the projections of the adult United Kingdom population with caries and periodontal diseases. Also, the incidence of caries and periodontal diseases and the transition to severe periodontal diseases were assumed to be the same across different age cohorts. Lastly, it is essential to emphasize that a global sensitivity analysis, which requires Monte Carlo simulations, was not conducted in this study. Future studies should consider performing this analysis to enhance the robustness of the uncertainties around the projections.

### Recommendations

The findings from this study that the older adult population (≥60 years old) is expected to experience the highest burden of carious teeth and periodontal diseases, while a significant decrease in burden is projected from individuals between the ages of 16 and 59 years, dictate the need for a radically different proactive approach to oral health to tackle this impended challenge. These efforts need to be undertaken alongside broader strategies to better align health systems with the population needs and avert age-related inequalities gaps. These projections also suggest that there are large rooms for improvement in dental caries and periodontal diseases prevention and control in the United Kingdom. We recommend that policymakers prioritize improving oral health in older adults by reorienting healthy aging policies to give greater attention to this area (52), particularly given the challenges faced by the struggling health system.

## Data availability statement

Publicly available datasets were analyzed in this study. This data can be found here: SN 6884—Adult Dental Health Survey, 2009 (Internet). 2009 (cited October 7, 2022). Available at: https://ukdataservice.ac.uk/find-data/.

## Ethics statement

All methods were carried out in accordance with relevant guidelines and regulations. Original ethical approval for Adult Dental Health Survey (2009) was obtained from the Oxford National Health Service (NHS) Research Ethics Committee for the survey to be conducted and anonymized, individual-level data are freely accessible to registered researchers via the United Kingdom Data Service. Anonymized data are accessible via the UK Data Archive for which no additional ethical approval was required.

## Author contributions

AE conceived the study. AE and JA designed the study and conducted the analysis and manuscript writing. JA developed the multi-state population model. All authors contributed to the article and approved the submitted version.

## Funding

This research was supported by Institute of Life Course Development, Centre for Chronic Illness and Aging, School of Human Sciences, Faculty of Education, Health and Human Sciences, University of Greenwich, London, United Kingdom.

## Conflict of interest

The authors declare that the research was conducted in the absence of any commercial or financial relationships that could be construed as potential conflict of interest.

## Publisher’s note

All claims expressed in this article are solely those of the authors and do not necessarily represent those of their affiliated organizations, or those of the publisher, the editors and the reviewers. Any product that may be evaluated in this article, or claim that may be made by its manufacturer, is not guaranteed or endorsed by the publisher.
