# Supplementary material for: Projecting the burden of dental caries and periodontal diseases among the adult population in the United Kingdom using a multi-state population model
Source: Front Public Health. 2023 Sep 7;11:1190197. doi: 10.3389/fpubh.2023.1190197 (PMC10513470; doi:10.3389/fpubh.2023.1190197)
Supplement: Supplementary file 1 [file Table_1.DOCX]

Appendix A: Model input table

| **Input name** | **Value/unit** | | | | | **Source** |
| --- | --- | --- | --- | --- | --- | --- |
| **Demographic input:** |  | | | | |  |
| Sex of babies |  | | | | |  |
| Female | 0.51 (dimensionless) | | | | | Office of National Statistics |
| Male | 0.49 (dimensionless) | | | | | Office of National Statistics |
| Fertility rate | [(2009,0.04), (2020,0.07)],(2009,0.059375),(2010,0.060625),(2011,0.0603125),(2012,0.060625),(2013,0.0578125),  (2014,0.0571875),(2015,0.056875),(2016,0.0565625),(2017,0.055),(2018,0.053125),(2019,0.0515625),(2020,0.049375)  (dimensionless/year) | | | | | Office of National Statistics |
| Age-specific mortality rate [Female] (newborn, age0-age100) | 0.004251 0.004251 0.000299 0.000194 0.000161 0.000121 9.9e-05 8.7e-05 8.3e-05 7.4e-05 9.6e-05 9e-05 9.3e-05 0.0001 0.000109 0.000117 0.000145 0.000177 0.000227 0.000261 0.000252 0.000239 0.00027 0.000249 0.00025 0.000266 0.000277 0.000306 0.000301 0.000329 0.000381 0.000403 0.000417 0.00049 0.000506 0.000592 0.00061 0.000613 0.000707 0.000782 0.000875 0.001003 0.001061 0.001122 0.001239 0.001344 0.001525 0.001628 0.001769 0.001962 0.002103 0.002493 0.00255 0.00278 0.003132 0.003549 0.003723 0.00405 0.004359 0.004704 0.005269 0.005648 0.006308 0.006648 0.007693 0.008288 0.008963 0.009933 0.010936 0.012061 0.013377 0.015009 0.016222 0.01803 0.020455 0.022841 0.0253 0.028753 0.032037 0.036131 0.041499 0.047283 0.052938 0.059318 0.067397 0.075803 0.085271 0.095864 0.108273 0.119017 0.132696 0.152698 0.170636 0.193597 0.213721 0.237529 0.264774 0.292157 0.314242 0.355837 0.376406 0.412248 (dimensionless/year) | | | | | Office of National Statistics |
| Age-specific mortality rate [Male] (newborn, age0-age100) | 0.005247 0.005247 0.000364 0.000218 0.000155 0.00012 0.000124 0.000113 9.3e-05 0.000118 9.8e-05 8.8e-05 0.000105 0.000111 0.000139 0.000164 0.00025 0.000332 0.000506 0.000597 0.000644 0.000696 0.000684 0.00068 0.000708 0.000722 0.000728 0.000802 0.000767 0.000836 0.000824 0.000913 0.000943 0.000995 0.001079 0.001159 0.001305 0.001263 0.001313 0.00145 0.001535 0.001667 0.001782 0.001923 0.002033 0.002142 0.002388 0.002533 0.002743 0.002927 0.003225 0.00356 0.003936 0.004271 0.004789 0.005141 0.005851 0.00628 0.006731 0.00746 0.00809 0.008644 0.009582 0.010496 0.011934 0.01304 0.014219 0.015839 0.01725 0.019573 0.021232 0.02275 0.025257 0.028202 0.031128 0.034262 0.038687 0.043238 0.047816 0.053019 0.060029 0.067381 0.075599 0.084213 0.093146 0.104787 0.116301 0.130939 0.145571 0.155993 0.170411 0.190027 0.207786 0.229398 0.251546 0.269897 0.313848 0.336461 0.376767 0.398299 0.428528 0.467846 (dimensionless/year) | | | | | Office of National Statistics |
| Net migration rate | 0.0052 (dimensionless/year) | | | | | Calibration |
| Initial Population of the UK [Female] (newborn, age0-age100) | 0 382112 384993 370847 363804 350792 344723 333592 327974 336117 343599 354963 360314 370396 366733 367822 379426 382918 398749 406963 402136 403019 414773 413629 420495 427041 419501 423250 425414 431069 432196 413624 388350 381636 392855 402635 411119 430120 450579 466932 456500 469218 469364 475339 477531 481863 477502 468255 456950 442075 425099 416519 408038 394077 381096 368366 370644 365944 358688 362159 371826 381354 404554 435948 342430 334338 333026 316097 286216 263087 273796 273395 267910 259085 250689 241795 229623 223787 222598 218204 209513 195825 182673 173487 164438 152582 141812 131223 122950 115052 95705 61238 49913 45770 39615 32703 25335 18791 13024 8806 5976 9649 (person) | | | | | Office of National Statistics |
| Initial Population of the UK [Male] (newborn, age0-age100) | 0 400614 402969 389095 379857 367838 361952 351164 344137 350766 361421 372366 377434 388510 388733 386599 398947 399088 409294 422883 416476 408564 412834 402912 415723 427425 417678 418898 417125 428581 433025 416963 390379 385159 391629 398580 404896 423555 442157 455774 449263 460484 462021 470860 467213 469274 464710 456286 447425 434508 418805 410675 403139 388351 375359 361930 363166 358803 349460 352403 359123 369849 390865 421411 329205 319185 318441 297867 266697 242181 251195 248534 242516 230670 219484 208906 193519 184241 178596 168977 155799 141915 127893 117745 106590 93338 82011 70795 63096 53147 42192 27140 20432 17432 13913 10690 7545 5087 3120 2047 1212 1475 (person) | | | | | Office of National Statistics |
| **Caries sub-model** |  | | | | |  |
| Incidence of caries rate | 0.0042 (dimensionless/year) | | | | | Calibration |
| Caries treatment uptake rate | 0.005 (dimensionless/year) | | | | | Calibration |
| Treatment cessation rate | 0.001 (dimensionless/year) | | | | | Calibration |
| Prevalence of caries   \| **Age** \| \| --- \| \| 16-24 \| \| 25-34 \| \| 35-44 \| \| 45-54 \| \| 55-64 \| \| 65-74 \| \| 75-84 \| \| 85 and over \| | Percentage (dimensionless)   \| 30 \| \| --- \| \| 36 \| \| 30 \| \| 26 \| \| 26 \| \| 22 \| \| 35 \| \| 28 \| | | | | | 2009 Adult Dental Health Survey |
| Fraction of the caries population treated | 0.84 (dimensionless) | | | | | 2009 Adult Dental Health Survey |
| **Periodontal disease sub-model** |  | | | | |  |
| Incidence 4mm rate | 0.0154 (dimensionless/year) | | | | | Calibration |
| From 4mm to 6mm rate | 0.0006 (dimensionless/year) | | | | | Calibration |
| From 6mm to 9mm rate | 0.0124 (dimensionless/year) | | | | | Calibration |
| Prevalence of periodontal diseases   \| **Age** \| \| --- \| \| 16-24 \| \| 25-34 \| \| 35-44 \| \| 45-54 \| \| 55-64 \| \| 65-74 \| \| 75-84 \| \| 85 and over \| | 4mm or more (dimensionless)   \| 19 \| \| --- \| \| 36 \| \| 43 \| \| 52 \| \| 61 \| \| 60 \| \| 61 \| \| 47 \| | | 6mm or more (dimensionless)   \| 1 \| \| --- \| \| 4 \| \| 7 \| \| 10 \| \| 16 \| \| 14 \| \| 14 \| \| 14 \| | | 9mm or more (dimensionless)  -   \| 0 \| \| --- \| \| 1 \| \| 2 \| \| 3 \| \| 3 \| \| 2 \|   - | 2009 Adult Dental Health Survey |
| **Loss of attachment sub-model** |  | | | | |  |
| Incidence 4mm LOA rate | 0.160 (dimensionless/year) | | | | | Calibration |
| From 4mm to 6mm LOA rate | 0.0172 (dimensionless/year) | | | | | Calibration |
| From 6mm to 9mm LOA rate | 0.002 (dimensionless/year) | | | | | Calibration |
| Prevalence of LOA   \| **Age** \| \| --- \| \| 55-64 \| \| 65-74 \| \| 75-84 \| \| 85 and over \| | 4mm or more (dimensionless)   \| 61 \| \| --- \| \| 67 \| \| 76 \|   72 | 6mm or more (dimensionless)   \| 18 \| \| --- \| \| 22 \| \| 25 \|   30 | | 9mm or more (dimensionless)   \| 2 \| \| --- \| \| 5 \| \| 5 \|   6 | | 2009 Adult Dental Health Survey |
