# Supplementary material for: Projecting the burden of dental caries and periodontal diseases among the adult population in the United Kingdom using a multi-state population model
Source: Front Public Health. 2023 Sep 7;11:1190197. doi: 10.3389/fpubh.2023.1190197 (PMC10513470; doi:10.3389/fpubh.2023.1190197)
Supplement: Supplementary file 2 [file Table_2.DOCX]

Appendix B: Comparing selected simulated outcome variables to data.
