# Supplementary figures and images for: Projecting the burden of dental caries and periodontal diseases among the adult population in the United Kingdom using a multi-state population model
Source: Front Public Health. 2023 Sep 7;11:1190197. doi: 10.3389/fpubh.2023.1190197 (PMC10513470; doi:10.3389/fpubh.2023.1190197)

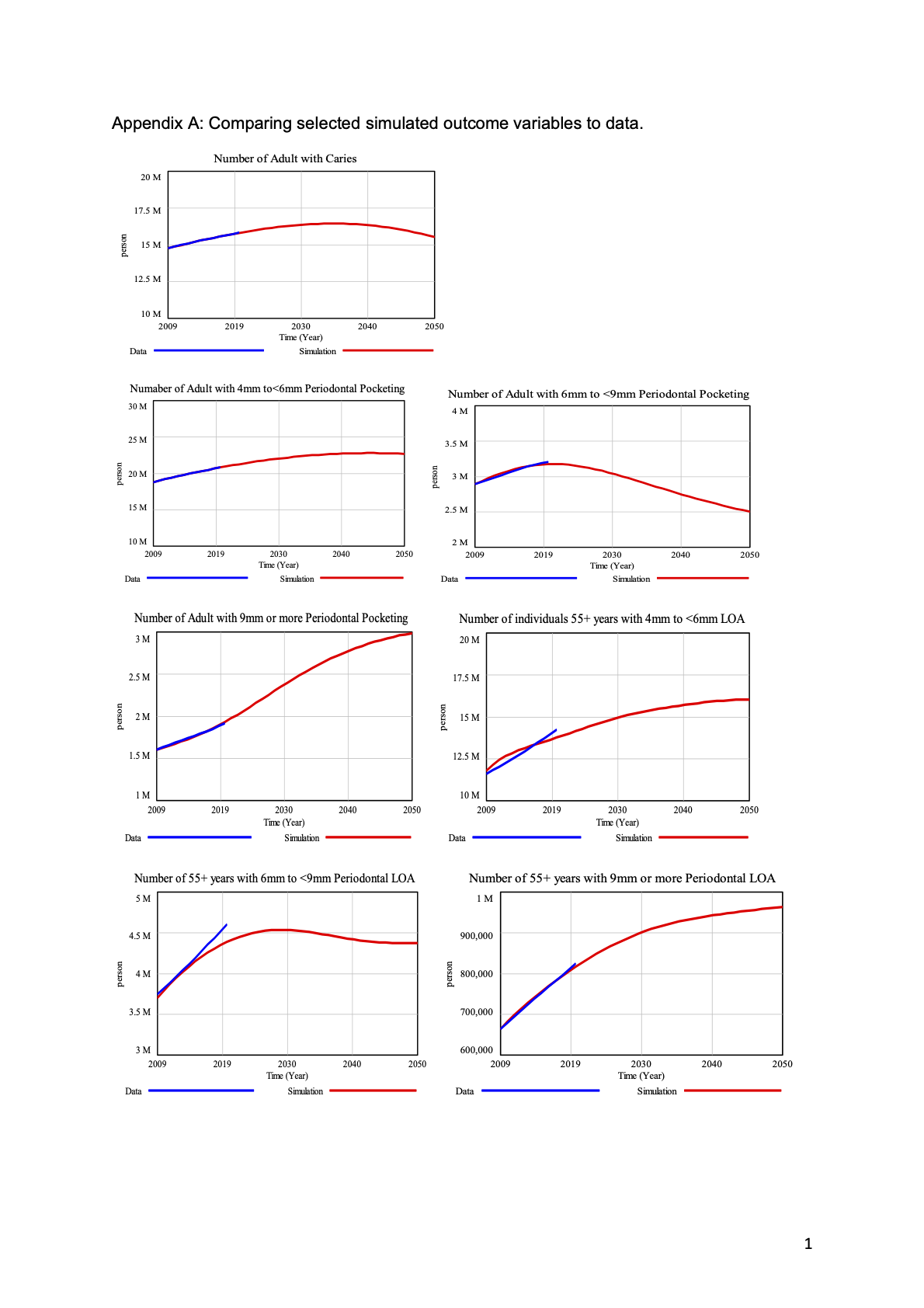

Supplement: Supplementary file 3 [file Image_1.TIFF]
